# A Positive Relationship between Exposure to Heavy Metals and Development of Chronic Diseases: A Case Study from Chile

**DOI:** 10.3390/ijerph18041419

**Published:** 2021-02-03

**Authors:** Sandra Cortés, Liliana Zúñiga-Venegas, Floria Pancetti, Alejandra Covarrubias, Muriel Ramírez-Santana, Héctor Adaros, Luis Muñoz

**Affiliations:** 1 Departamento de Salud Pública, Escuela de Medicina, Universidad Pontificia Universidad Católica de Chile, Santiago 8330077, Chile; 2 Advanced Center for Chronic Diseases (ACCDIS), Santiago 8330077, Chile; 3 Centro de Desarrollo Urbano Sustentable (CEDEUS), Santiago 8330077, Chile; 4 Laboratorio de Investigaciones Biomédicas (LIB), Departamento de Preclínicas, Facultad de Medicina, Universidad Católica del Maule, Talca 3480005, Chile; lzuniga@ucm.cl; 5 Centro de Investigaciones y Estudios Avanzados del Maule (CIEAM), Universidad Católica del Maule, Talca 3480005, Chile; 6 Laboratorio de Neurotoxicología Ambiental, Departamento de Ciencias Biomédicas, Facultad de Medicina, Universidad Católica del Norte, Larrondo No. 1281, Coquimbo 1781421, Chile; pancetti@ucn.cl (F.P.); alejandra.covarrubias@ucn.cl (A.C.); 7 Centro de Investigación y Desarrollo Tecnológico en Algas y Otros Recursos Biológicos, Universidad Católica del Norte, Larrondo No. 1281, Coquimbo 1781421, Chile; 8 Departamento de Salud Pública, Facultad de Medicina, Universidad Católica del Norte, Coquimbo 1781421, Chile; mramirezs@ucn.cl; 9 Hospital Dr. Jerónimo Méndez, Chañaral, Chañaral 1490000, Chile; hectoradarosm@gmail.com; 10 Laboratorio de Metrología Química, Comisión Chilena de Energía Nuclear, Santiago 7600713, Chile; luismunoza1954@gmail.com

**Keywords:** metabolic disorders, metal exposure, glycemia, cholesterol

## Abstract

Chile is a mining country, where waste mining is frequently found in the vicinity of inhabited areas. To explore the association between metal exposure and alterations in glucose metabolism, inflammatory status, and oxidative stress in individuals with chronic exposure to metals, a cross-sectional study was performed with 25 volunteers, between 45–65 years old. Inductive coupled plasma mass spectrometry (ICP-MS) was used to measure urinary levels of total arsenic (As) and its metabolites, cooper, nickel, chromium, and lead. Lipid profile, glucose, and insulin were measured in blood, as well as inflammation (interleukin-6, IL-6) and oxidative stress (8-hydroxy-2′deoxyguanosine, 8-OHdG) markers. Increased levels of Low-density lipoprotein, high-density lipoproteins, cholesterol and 8-OHdG, and the index for homeostasis model assessment—insulin resistance (HOMA-IR) were observed in 72%, 60%, and 56% of the volunteers, respectively. Blood-glucose levels were correlated with dimethylarsinic acid (DMA) (R^2^ = 0.47, *p* = 0.019), inorganic As (As_i_) (R^2^ = 0.40, *p* = 0.012), and Ni (R^2^ = 0.56; *p* = 0.044). The models with these compounds explained 72% of the glycemia variability (β_DMA_ = −6.47; β_Asi_ = 6.68; β_Ni_ = 6.87). Ni showed a significantly influence on IL-6 variability (β = 0.85: R^2^ = 0.36). Changes in glycemia could be related to exposure to low levels of As_i_ and Ni, representing risk factors for metabolic diseases. Body mass index would confuse the relation between IL-6 and Ni levels, probably due to known chronic inflammation present in obese people.

## 1. Introduction

Mining waste contains high concentrations of chemical products, often being stored in dams or reservoirs, causing an important source of contamination by inorganic chemical elements [1,2]. Among these, Pb, Cr, Cd, Cu, Zn, Hg and Ni, as well as As metalloid are of special interest due to their abundance in mining waste and impact on human health [3]. In developing countries, exposure to metals from activities related to metal mining constitutes a potential health risk for the general population. Furthermore, possible exposure to heavy metals by food consumption also exists. The evidence indicates that exposure to metals has an impact on respiratory health [4] and has been linked to lung cancer [5], neurodevelopmental disorders [6], and cardiovascular disorders [5,7], among other pathologies. The effects depend on the elements to which the communities are exposed to and the characteristics of the exposure [7].

In Chile, a country whose gross domestic product (GDP) strongly depends on mining activity, a total of 726 mining waste deposits have been identified [8]. Additionally, 530 million tons are generated annually, accumulating a total of 23 billion tons in the national territory. The Atacama region, located 900 km north of the Chilean capital, Santiago, stores more than 22% of the country’s mining waste [9]. Within it, the Chañaral Bay was recognized in 1983 by the United Nations Environment Program (UNDP) as an area with the most serious mining pollution problems in the world [10]. Despite the damage to the biotic ecosystem that has been extensively reported [11,12,13,14], there is no information about the impact on human populations. In a previous study, the total and inorganic As urinary concentrations in a sample of adults from Chañaral were measured, which exceeded international recommendations for both compounds (95th percentile) (232.2 μg/L and 63.2 μg/L, respectively, compared with 50.0 μg/L as reference) [15]. The same tendency was observed for the rest of metals in urine: Ni (7.2 μg/L versus 4.1 μg/L), Cu (41.9 μg/L versus 13.0 μg/L), Hg (6.3 μg/L versus 4.0 μg/L), and Pb (4.5 μg/L versus 2.6 μg/L) measurements [15].

The lifestyle, diet, and aging of the population have led to an increase in chronic diseases worldwide. Particularly in Chile, there is an increase in the prevalence of metabolic diseases according our national health surveys [16], and it is known that the imbalance between reactive oxygen species (ROS) and the antioxidant mechanisms of an individual has an impact on the metabolism of lipids and carbohydrates. Furthermore, the inflammatory state and oxidative stress also have a role in the pathogenesis of metabolic diseases. The oxidation of biomolecules is usually accompanied by a generalized inflammatory response, followed by the development of diseases associated with the formation of free radicals [17]. One of the main targets of oxidative stress, in addition to lipids and proteins, is the DNA molecule, increasing the probability of mutagenic events. The main product of DNA oxidation is 8-hydroxyideoxyguanosine (8-OHdG) [18], a metabolite that is widely used for the evaluation of oxidative stress. The inflammatory stimulus produced by ROS, results in the synthesis and secretion of cytokines initiating the inflammatory response. It has been described that a chronic state of inflammation is involved in the pathogenesis of several diseases such as insulin resistance, type 2 diabetes mellitus, metabolic syndrome, and atherosclerosis, among others [19]. Some of the biomarkers of inflammatory damage associated with exposure to contaminants are interleukins of type 6, 8, and 10 (IL-6, IL-8, IL-10) [17].

To the best of our knowledge, no studies have been conducted in Chile on the association between heavy metal exposure and the above health markers. The objective of this article was to explore the association between chronic exposure to metals and alterations in biochemical parameters associated with glucose metabolism, inflammatory status, and oxidative stress, in residents from the urban area of Chañaral Bay, Chile, chronically exposed to metals.

## 2. Material and Methods

### 2.1. Design

The results shown in this report were obtained from a larger study carried out in the city of Chañaral, whose objective was to establish the exposure to metals after a large flood that occurred in March 2015. In that case, the main study was carried out under the approval of the committee of ethics of the School of Medicine (# 15-089, 15 June 2015). An exploratory cross-sectional study was performed. Adult volunteers, ages 45 to 65, and with uninterrupted residence in the city of Chañaral for the past 5 years were invited to participate [20].

### 2.2. Sampling

Among the participants from the bigger study, a subsample was identified. Volunteers with a medical diagnosis of autoimmune pathologies and allergies, thyroid abnormalities, diabetes, hypercholesterolemia, and insulin resistance were excluded to avoid confusion bias. Each participant was asked to sign an informed consent and was interviewed at their home by trained personnel. Each volunteer answered a questionnaire regarding demographic characteristics (age, education), lifestyles (smoking, use of alcohol), metal exposure (eating fish and shellfish), and health status (chronic diseases self-reported), and donated a 50 mL urine sample at the time of the interview. All the procedures were previously used in the National Health Surveys [16,21]. The samples were stored immediately at 4 °C after being obtained and then were transferred to the laboratory of the Chañaral’s regional hospital where they were frozen. Samples were sent to Santiago, Chile, preserving the cold chain at −20 °C, to be analyzed at the Comisión Chilena de Energía Nuclear (CChEN) Metrology Laboratory.

### 2.3. Analysis of Metals and Metabolic Parameters

Urinary levels of total arsenic (As), inorganic arsenic (As_i_), As species including As(III), As(V), monomethylarsonic acid (MMA), dimethylarsinic acid (DMA), arsenobetaine (AsBet), Cu, Ni, Cr, and Pb were established in laboratories of the Chilean Nuclear Energy Commission (CCHEN), using the validated technique of ICP-MS. Limits of detection (LOD) for each metal were 0.35 µg/L. All samples exceeded LOD for total As and As_i_. On the other hand, 0% of samples did not exceed its LOD for MMA, copper, and lead.

Aside from urine, 10 mL of fasting blood samples were obtained by venipuncture. Lipid profile, insulin, and glycemia were measured in the local laboratory, using techniques widely used and validated in the national health surveys used in Chile [16,21,22].

Immunoassay techniques, ELISA (R&D Systems, Minneapolis, MN, USA) were used to measure inflammation and oxidative stress markers (IL-6 and 8-OHdG). For this, 1 mL plasma samples were quantified using human IL-6 ELISA kit, EH21IL6; INVITROGEN, (Thermo Fisher Scientific, Waltham, Massachusetts, USA) and oxidative DNA damage ELISA kit, STA-320 (Cell Biolabs, San Diego, California, USA). For both kits, 50 μL of the original sample was used. A calibration curve was carried out and the absorbance measurement was at 450 nm in the multifunctional microplate reader, NOVOstar 0700 (BMG Labtech, Ortenberg, Germany). These results were obtained in the Environmental Neurotoxicology laboratory of the Universidad Católica del Norte in Coquimbo, Chile.

### 2.4. Statistical Analysis

Univariate and bivariate descriptive statistical analyses were performed to determine the distribution of the damage and exposure parameters. Parametric variables were expressed as mean ± SD and comparisons were performed using *t*-Student test; on the contrary, non-parametric variables were expressed as median (interquartile range) and comparisons were performed using U Mann–Whitney test. For the biochemical parameters, normal values were then established according to the National Health Survey [16]: insulin (3–15 µU/mL), glycemia (70–99 mg/dL), total cholesterol (<200 mg/dL); triglycerides (<150 mg/dL), high-density lipoproteins (HDL) (>40♂; >50♀ mg/d/L), Low-density lipoprotein (LDL), (<100 mg/dL), index Castelli (<4.5), homeostasis model assessment (HOMA) (<2). Normal weight considers those with a BMI less than 30; the rest of participants are considered obese [16].

According to the manufacturers, the cut-off points were for IL-6 (median and range) 2.59 (0.89–7.28) (pg/mL), and for 8-OHdG (mean ± SD) 6.61 ± 0.18 ng/mL, respectively.

In those metals with values lower than the LOD, these were analyzed considering values given by the square root of the LOD divided by 2 for statistical analysis [23].

Correlations and multivariate linear regression models were also performed to establish the role of other variables. Hypothesis tests were proven with a significance level of 5% (*p*-value < 0.05). The statistical programs SPSS version 17.0 (IBM, Armonk, NY, USA) and R (R Core Team, Auckland, New Zealand) were used [24,25].

## 3. Results

### 3.1. Description of the Participants

In Table 1, the sociodemographic characteristics, metal levels, metabolic parameters, and early biological markers of the whole study group, separated by sex and weight, are described. The median age of participants was 48 years old and 76% of the sample were women. No statistical differences were found between women and men for any variable except for smoking frequency, in which 83% of men were smokers compared to 32% of women. On average, Table 1 shows that most of the biochemical/metabolic parameters were within normal ranges in this sample [16], except the body mass index indicating the predominance of obesity or overweight in the studied sample.

### 3.2. Associations between Biochemical Parameters and Measured Metals

**Figure 1:**
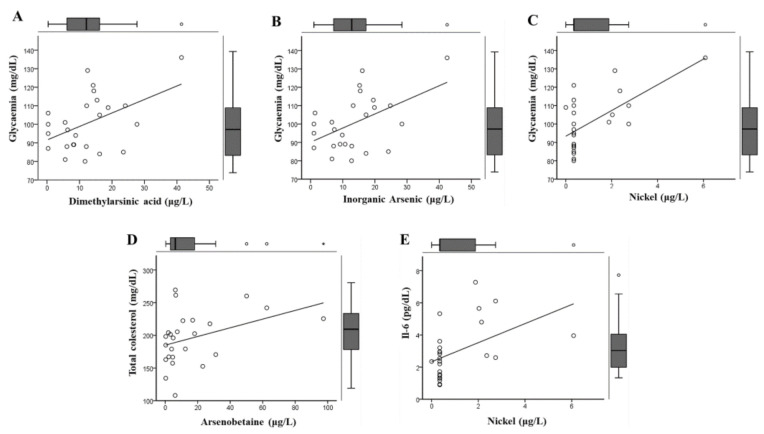
Significant correlations between biochemical and clinical parameters and levels of metals. The distributions of these parameters are shown using boxplots.

Significant correlations between biochemical and clinical parameters with levels of metals are presented in Figure 1. Glycemia was significantly correlated with DMA (R^2^ = 0.47, *p* = 0.019), As_i_ (R2 = 0.40, *p* = 0.012), and Ni (R^2^ = 0.56, *p* = 0.044).

Additionally, the dichotomization by each biochemical/metabolic parameter by individual between normal or abnormal values resulted in the distribution showed in Figure S1, which shows that more than 40% of the sample presented abnormal levels for all the biochemical/metabolic parameters and biological markers measured. The parameters that showed the highest percentages of affected individuals were LDL cholesterol (72% of the subjects over the normal value of 100 mg/dL), followed by HDL cholesterol (60% over the normal value), and 8-OHdG and the HOMA-IR index (56% each).

Table 2 shows the effect of the level of each metal on biochemical and clinical parameters. It is possible to see that individuals with normal glycemia did not have detectable levels of nickel, whereas individuals with altered glycemia showed a median of 1.88 µg/L of this metal, with 25% of subjects’ urine concentrations above 2.55 µg/L.

A similar pattern was observed for IL-6, where individuals with lower levels of inflammation did not show detectable levels of nickel (*p* = 0.022). It was possible to observe a tendency to altered glycemia in those individuals with highest levels of DMA and As_i_, however these differences were not significant (*p* = 0.083 and *p* = 0.069, respectively). A significant increase of AsBet level (3.06-fold) in subjects with altered total cholesterol was found (*p* = 0.016), and there is a clear tendency towards lower levels of AsBet in subjects with insulin resistance with respect to those with normal HOMA-IR index (*p* = 0.059). Individuals presenting higher levels of 8-OHdG also showed low levels of As_o_.

### 3.3. Effect of Other Covariates Associated with Early Damage Parameters

Finally, in order to determine whether age, sex, and BMI influenced the biochemical/metabolic parameters, irrespective of metal concentration, multivariate regression models were performed (Table 3). Female sex (β = 14.5), DMA (β = −6.47), As_i_ (β = 6.68), and Ni (β = 6.87), were jointly able to explain 72% of the glycemia variability (*p =* 0.002). Nickel showed a significantly influence on IL-6 variability (β = 0.85; *p* = 0.017). The remaining biochemical and clinical parameters were not significantly associated with any metals.

Supplementary Table S1 presents the regressions models without the BMI.

## 4. Discussion

The objective of this article was to evaluate the association between exposure to mining waste metals and alterations in biochemical parameters associated with glucose metabolism, inflammatory status, and oxidative stress, in residents of the urban area of Chañaral Bay, Chile; an area with historical exposure to metals.

### 4.1. Findings of this Study

This preliminary study shows that the metal levels in urinary samples were low. Especially, Cu and Pb were below their detection limits. The most relevant results were obtained for total and As_i_ with no differences by sex. These results obtained in this small sample are below other values from national studies [15,26].

Regarding biochemical parameters, those related to cholesterol were the most prevalently altered, especially LDL and HDL (72.0% and 60.0% of participants, respectively) exceeding other values measured in similar Chilean studies [27].

For As_i_ and Ni, differences were observed between high levels and altered glycemia (*p*-value = 0.069 and 0.022 respectively); AsBet levels showed significant differences with total cholesterol. In relation to the early biomarkers of inflammation and oxidative stress, it was only possible to establish a significant difference for high levels of IL-6 in people exposed to Ni. In adjusted models, the differences continued being significant for As_i_ and Ni when glycemia levels increased. However, this tendency vanished for total cholesterol and AsBet when adjusting by sex, age, and BMI. An interesting result was observed by introducing BMI as a variable in the models, considering that an important proportion of the studied individuals showed increased BMI. It turned out that obesity would be confusing the relation between IL-6 and nickel, by increasing the influence of nickel in the inflammatory status of individuals in over 40% (from 25% to 36%) when including BMI in the model. This is not rare, since obese people are proven to have a permanent increased inflammatory status [28]. On the other hand, the relation between metals and glycemia did not change much when including BMI in the regression model, neither with cholesterol.

### 4.2. Toxicological Implications

Among the toxicological implications of our findings, it is noteworthy that AsBet is a low toxicity organic arsenic compound, whose source of exposure is through diet. AsBet is abundant in fish, shellfish, and seaweed. Therefore, a possible explanation for the association between high levels of AsBet and high cholesterol could be a diet based on shellfish consumption. On the other hand, individuals who presented with HOMA-IR index in normal ranges had higher levels of AsBet in urine compared to individuals with altered HOMA index, these differences being close to statistical significance (*p* = 0.059). In other words, it could be considered that AsBet ingested through the seafood diet would be a protective factor for insulin resistance [29,30]. Similarly, high levels of AsBet in urine were found in those individuals with the lowest rate of oxidative damage in DNA (measured through plasma levels of 8-OHdG), which would indicate a protective effect that is probably due to the intake of nutritional components found in seafood products such as omega-3 fatty acids and vitamins A, D, and E [31]. It is known that As triggers diabetogenic effects [32,33]. Specifically, it has been determined that As inhibits insulin signaling through the PDK-1/PKB/Akt [34] transduction pathway in vitro. Our data show an inverse effect between the concentration of AsBet and the HOMA-IR index, but not between the concentration of DMA or As_i_ and glycemia. In this case, individuals who presented with altered glycemia also had a higher concentration of DMA and As_i_ in the urine. This association was not statistically significant individually (*p* = 0.083 and *p* = 0.069, respectively), probably because of the small sample studied. Nevertheless, the influence of metals in increasing the glycemia was significant when performing the regression model.

Finally, detectable levels of Ni were found in individuals with high glycemia and elevated levels of IL-6, a marker of inflammation [35]. Several studies have demonstrated the relationship between cytokine expression and the exposure to particulate material, both in vitro [36] and in vivo [37]. Interestingly, the association between high levels of Ni and elevated glycemia has also been reported previously in an study conducted in China [38]. Interaction with obesity in this association was already mentioned.

### 4.3. Limitations and Strengths for the Public Health

Given the exploratory nature of the study carried out, several limitations should be reported. Among them, its low sample size, with small number of men and a unique sample from the study area, does not allow for stratified analyses by sex or other variables such as smoking habits. Moreover, only two biological markers related to cellular damage were analyzed. Since these analyses were preliminary and due to the small sample size, we also do not correct for multiple comparisons.

Despite the limitations of an exploratory study, this report provides promising indicators of early damage in people with heavy metal exposure and advocates for the exploration of biomarkers related to metabolic alterations—including a broad spectrum of other early damage markers—in population-based studies. This kind of evidence will be critical for protecting populations chronically exposed to metals and other compounds, such as As, even those with currently low levels.

The results obtained in this pilot study will allow us to strengthen and expand our study of chronic diseases, especially those of a metabolic type, associated with environmental conditions, beyond lifestyles, diet, or genetic conditions. In developing countries, as is the case in Latin America, it is necessary to broaden the scope as there are high rates of chronic alterations together with exposure to a mix of pollutants. The results presented have generated an alert in our communities linked to mining activities. At the level of health public policies, we need to not only to measure their levels of metals by biomonitoring but also to establish associations with metabolic alterations at the population level and reduce the current exposures.

## 5. Conclusions

This exploratory analysis shows a positive association between urinary Ni levels and glycemia and the inflammation marker IL-6.

Despite the current low levels of metals and As, and their compounds, there is a positive association between urinary AsBet levels and total cholesterol, and between urinary inorganic arsenic levels and glycemia. There is a negative association between urinary AsBet and 8-OHdG.

The results from this small sample evidence a possible mechanism related to the occurrence of chronic diseases, especially among those related to alterations of glucose metabolism and chronic inflammation in people exposed to low levels of As_i_ and Ni. Both contaminants will be present in people from communities similar to Chañaral with respect to waste mining.

Interestingly, the exposure to AsBet, a compound considered safe in health risk assessment and associated with high total cholesterol, should be evaluated in future studies.

Finally, considering the importance of mining, and the exposure to metals and As in the general population of those residing close to mining facilities, exposure to metals and As is a risk factor that must be explored together with health effects related to metabolic diseases.

## Figures and Tables

**Table 1:** Characteristics of the study sample, Chañaral, Chile. Statistical analysis between sex and between normal weight and obese individuals.

Variable	Total *(n* = 25)	Women (*n* = 19)	Men (*n* = 6)	Healthy Weight (*n* = 13)	Obese (*n* = 9) *
Socio demographic					
Age (years)	48.00 (45.50–53.00)	48.00 (46.00–51.00)	50.50 (43.75–54.25)	49.0 (44.0–54.5)	47.0 (44.5–51.5)
Weight (kg) ^~^	78.9 ± 17.0	75.8 ± 17.3	87.0 ± 14.4	70.0 (57.5–79.0)	90.0 (81.0–104.0)
Height (cm)	160.5 ± 8.7	156.7 ± 5.9	170.9 ± 7.0	158.0 (152.5–167.0)	158.0 (156.5–166.5)
Body mass index (kg/m^2^) ^~^	30.4 ± 5.0	30.6 ± 5.4	29.7 ± 4.2	27.27 (24.72–29.03)	35.30 (33.73–37.47)
Time living in the area (years)	42.00 (29.50–46.00)	45.00 (30.00–46.00)	32.50 (24.25–43.50)	42.00 (29.50–46.50)	46.0 (29.50–47.00)
Time living in the current house (years)	17.92 ± 10.90	18.53 ± 10.87	16.00 ± 11.08	12.0 (10.0–12.0)	12.0 (11.5–12.0)
Study level (years of instruction)	11.7 ± 2.2	11.3 ± 1.5	13.2 ± 3.2	20.00 (8.00–24.00)	16.00 (13.50–27.50)
Smokers (*n*, %)	10 (44)	6 (32)	4 (67)	6 (46)	3 (33)
Alcohol drinkers (*n,* %)	6 (24)	2 (12)	4 (67)	4 (31)	2 (22)
Metal levels					
As(III) (µg/L)	0.35 (0.35–0.35)	0.35 (0.35–0.35)	<LOD	0.35 (0.35–0.35)	0.35 (0.35–0.35)
As(V) (µg/L)	0.35 (0.35–0.35)	0.35 (0.35–0.35)	<LOD	0.35 (0.35–0.35)	0.35 (0.35–0.35)
Monomethylarsonic acid (µg/L)	<LOD	<LOD	<LOD	< LOD	< LOD
Dimethylarsinic acid (µg/L)	12.41 ± 9.63	12.44 ± 9.82	12.32 ± 9.88	12.10 (4.24–17.5)	6.35 (5.58–13.95)
Inorganic arsenic (µg/L)	13.66 ± 9.67	13.78 ± 9.88	13.27 ± 9.86	12.80 (5.29–18.55)	7.4 (6.63–16.67)
Arsenobetaine (µg/L)	6.25 (2.72–20.60)	6.13 (3.21–23.10)	9.33 (0.35–19.55)	4.91 (2.11–22.20)	6.49 (2.07–15.25)
Total arsenic (µg/L)	10.70 (5.68–30.10)	10.70 (6.14–30.40)	9.84 (4.85–19.80)	10.70 (5.90–30.95)	9.42 {3.35–21.55)
Chromium (µg/L)	0.35 (0.35–0.35)	0.35 (0.35–0.35)	0.35 (0.35–2.19)	0.35 (0.35–0.35)	0.35 (0.35–0.35)
Nickel (µg/L)	0.35 (0.35–1.96)	0.35 (0.35–2.14)	0.35 (0.35–0.70)	0.35 (0.35–0.35)	0.35 (0.35–2.00)
Copper (µg/L)	<LOD	<LOD	<LOD	< LOD	< LOD
Lead (µg/L)	<LOD	<LOD	<LOD	<LOD	< LOD
Biochemical parameters					
Basal insulin (µU/mL) ˠ	15.84 ± 8.68	14.63 ± 7.72	19.68 ± 11.09	12.30 (7.90–12.30)	19.50 (14.05–28.95)
Glycaemia (mg/dL)	100.60 ± 15.02	98.79 ± 15.89	106.33 ± 11.06	95.00 (87.50–110.00)	100.00 (91.00–109.00)
Total cholesterol (mg/dL)	195.66 ± 39.77	193.14 ± 38.83	203.45.46	185.00 (161.95–220.35)	201.40 (164.95–204.75)
Triglyceride (mg/dL)	127.50 (93.00–218.00)	125.10 (84.50–203.40)	243.20 (133.45–370.68)	125.10 (71.15–194.55)	211.50 (130.20–261.30)
HDL (mg/dL)	47.02 ± 8.28	48.44 ± 8.39	42.53 ± 6.63	47.80 (44.00–49.15)	45.00 (41.00–51.50)
LDL (mg/dL)	118.89 ± 36.00	120.58 ± 39.38	113.53 ± 24.34	109.10 (98.00–133.45)	106.20 (87.55–129.15)
Castelli index	4.25 ± 1.04	4.04 ± 0.86	4.88 ± 1.38	3.7 (3.35–4.55)	3.9 (3.75–4.85)
HOMA index	3.98 ± 2.22	3.58 ± 1.89	5.26 ± 2.88	2.90 (1.61–4.33)	5.64 (3.31–7.34)
Clinical parameters					
IL-6 (pg/mL)	2.59 (1.50–3.78)	2.83 (1.55–3.96)	2.27 (1.33–3.21)	2.35 (1.38–3.45)	3.20 (1.52–5.23)
8-OHdG (ng/mL)	6.61 ± 0.18	6.63 ± 0.20	6.56 ± 0.13	6.62 (6.58–6.77)	6.53 (6.43–6.80)

Parametric variables are expressed as mean ± SD, and non-parametric variables as median (interquartile range). ^~^ *U* Mann–Whitney test *p*-value < 0.001. ˠ *U* Mann–Whitney test *p*-value = 0.021. HDL—high-density lipoproteins. LOD—Limits of detection. LDL—Low-density lipoprotein.* 3 participants had data loss of weight or height.

**Table 2:** Differences in heavy metal concentrations on people with normal vs. altered biochemical and biomarkers levels.

Variable	Dimethylarsenate (µg/L)	Inorganic Arsenic (µg/L)	Arsenobetaine (µg/L)	Total Arsenic (µg/L)	Nickel (µg/L)
Basal insuline (µU/mL)					
Normal	13.20 ± 11.20	14.25 ± 11.16	6.19 (3.72–35.85)	10.45 (5.03–36.63)	0.35 (0.35–0.95)
Altered	11.44 ± 7.56	12.91 ± 7.83	7.41 (1,92–16.90)	11.40 (6.14–19.10)	0.35 (0.35–2.03)
*p*-value	0.661 ^a^	0.739 ^a^	0.609 ^b^	0.647 ^b^	0.767 ^b^
Glycemia (mg/dL)					
Normal	8.96 ± 6.44	10.01 ± 6.45	4.77 (2.24–27.85)	8.42 (3.96–30.25)	<LOD
Altered	15.63 ± 11.13	17.03 ± 11.10	7.41 (3.38–20.00)	12.70 (6.56–29.75)	1.88 (0.35– 2.55)
*p*-value	0.083 ^a^	0.069 ^a^	0.728 ^b^	0.376 ^b^	0.022 ^b^
Total, cholesterol (mg/dL)					
Normal	12.10 (0.35–15.80)	12.55 ± 11.31	4.53 (0.35–9.27)	9.42 (5.18–21.55)	0.35 (0.35 - 2.09)
Altered	12.95 (6.19–22.33)	14.87 ± 7.83	13.90 (6.31–44.45)	15.90 (5.44–31.68)	0.35 (0.35–1.50)
*p*-value	0.406 ^b^	0.560 ^a^	0.016 ^b^	0.437 ^b^	0.769 ^b^
Triglyceride (mg/dL)					
Normal	12.57 ± 11.24	13.58 ± 11.24	4.91 (3.21–31.10)	10.70 (4.48–36.1)	0.35 (0.35–2.36)
Altered	12.21 ± 7.06	13.78 ± 7.26	9.91 (2.15–19.25)	11.06 (5.91–28.15)	0.35 (0.35–1.92)
*p*-value	0.928 ^a^	0.960 ^a^	0.723 ^b^	0.849 ^b^	0.849 ^b^
HDL (mg/dL)					
Normal	10.26 (0.35–20.13)	12.12 ± 9.96	11.58 (0.35–33.15)	8.96 (2.20–30.83)	< LOD
Altered	12.20 (6.35–10.20)	14.68 ± 9.68	6.13 (3.92–12.40)	10.70 (6.64–27.40)	0.35 (0.35–2.14)
*p*-value	0.643 ^b^	0.528 ^a^	0.978 ^b^	0.428 ^b^	0.261 ^b^
LDL (mg/dL)					
Normal	14.04 ± 13.26	15.74 ± 14.00	4.62 (0.35–12.40)	11.40 (3.78–27.40)	0.35 (0.35–2.14)
Altered	11.80 ± 7.89	12.85 ± 7.79	6.95 (3.74–28.40)	10.45 (5.91–30.83)	0.35 (0.35–0.73)
*p*-value	0.611 ^a^	0.513 ^a^	0.220 ^b^	1.000 ^b^	0.458 ^b^
Castelli index (mg/dL)					
Normal	11.90 ± 19.32	13.35 ± 10.53	4.77 (1.75–24.20)	11.05 (5.88–30.83)	0.35 (0.35–0.80)
Altered	13.10 ± 9.08	14.05 ± 8.94	7.41 (3.21–16.90)	10.200 (5.21–19.10)	0.35 (0.35–2.03)
*p*-value	0.764 ^a^	0.861 ^a^	0.467 ^b^	0.767 ^b^	0.687 ^b^
HOMA index					
Normal	14.62 ± 10.05	15.73 ± 9.83	40.60 (3.77–71.23)	32.95 (5.88–44.28)	0.35 (0.35–0.95)
Altered	11.73 ± 9.65	13.01 ± 9.79	6.13 (2.22–12.40)	10.20 (5.21–19.10)	0.35 (0.35–2.03)
*p*-value	0.533 ^a^	0.558 ^a^	0.059 ^b^	0.176 ^b^	0.687 ^b^
IL-6 (µg/mL)					
Below median	9.03 (1.66–18.15)	11.57 ± 8.72	6.37 (0.74–30.20)	15.24 ± 14.38	< LOD
Above median	12.20 (8.20–15.80)	15.59 ± 10.43	6.13 (4.23–15.25)	21.25 ±21.25	1.88 (0.35–2.55)
*p*-value	0.470 ^b^	0.309 ^a^	0.979 ^b^	0.406 ^a^	0.022 ^b^
8-OHdG (ng/mL)					
Below mean	15,15 ± 6.68	16.65 ± 6.58	12.40 (6.25–27.50)	15.70 (6.14–29.80)	0.35 (0.35–2.14)
Above mean	10.28 ± 11.19	11.31 ± 11.21	4.58 (0.35–9.39)	8.59 (4.31–31.83)	0.35 (0.35–0.35)
*p*-value	0.216 ^a^	0.175 ^a^	0.058 ^b^	0.501 ^b^	0.291 ^b^

^a^ Parametric variables are expressed as mean ± SD; *p*-values were calculated performing *t*-Student test. ^b^ Non-parametric variables are expressed as median (interquartile range); *p*-values were calculated performing *U* Mann–Whitney test. LOD—Limit of detection.

**Table 3:** Associations among chronic exposure to metals and glycemia, IL6, and cholesterol levels, in Chilean people, adjusted by age, body mass index, and sex (linear regression models).

Dependent Variable	Explanatory Variables	Coefficients			Model		
		β	Standard Error	*p*-Value	R^2^	Adjusted R^2^	*p*-Value
Glycemia	Age (years)	−0.49	0.40	0.904	0.72	0.60	0.002
	Sex	14.50	5.06	0.012			
	BMI	1.93	4.85	0.686			
	Dimethylarsinic acid (µg/L)	−6.47	2.73	0.032			
	Inorganic arsenic (µg/L)	6.68	2.65	0.024			
	Nickel (µg/L)	6.87	2.37	0.011			
IL-6	Age (years)	−0.05	0.06	0.419	0.36	0.21	0.088
	Sex	−0.004	0.83	0.999			
	BMI	0.81	0.75	0.295			
	Nickel (µg/L)	0.85	0.32	0.017			
Cholesterol	Age (years)	−1.88	1.21	0.137	0.28	0.11	0.214
	Sex	20.91	19.06	0.288			
	BMI	8.80	17.70	0.625			
	Arsenobetaine (µg/L)	1.10	0.66	0.11			

For sex variable, women were the reference group. For BMI variable, normal weight was the reference group.

## Data Availability

Data are available on request to the corresponding author considering that the preliminary results presented in this report are part of a pilot study. In this way, our results are not generalizable for the whole population of Chañaral.

## Supplementary Materials

The following are available online at https://www.mdpi.com/1660-4601/18/4/1419/s1, Figure S1: Percentages of individuals with normal and altered biochemical parameters according to the National Health Survey [16,21], or above and below of measurements of central tendency, according to their distribution, of the clinical parameters measured (median for IL-6: mean for 8-OHdG). See Table S1 for the dispersion values of the clinical parameters, Table S1: Linear regression models for the biochemical parameters which were statistically influenced by some metals, adjusted by age and sex.
