# Supplementary material for: A Positive Relationship between Exposure to Heavy Metals and Development of Chronic Diseases: A Case Study from Chile
Source: Int J Environ Res Public Health. 2021 Feb 3;18(4):1419. doi: 10.3390/ijerph18041419 (PMC7913508; doi:10.3390/ijerph18041419)
Supplement: Supplementary file 1 [file ijerph-18-01419-s001.pdf]

Supplementary Table S1.

Linear regression models for the biochemical parameters which were statistically influenced by some metals, adjusted by age and sex.

Dependent variable	Explanatory variables	Coefficients			Model		
		β	Standard error	*p*-value	R^2	Adjusted R^2	*p*-value
Glycemia	Age (years)	-0.78	0.37	0.836	0.70	**0.62**	**<0.001**
	Sex (male)	15.33	4.67	0.004			
	Dimethylarsinic acid (μg/L)	-7,34	2,32	0.005			
	**Inorganic arsenic (μg/L)**	**7,37**	**2,31**	**0.005**			
	**Nickel (μg/L)**	**7,90**	**2,04**	**0.001**			
IL-6	Age (years)	-0.05	0.06	0.447	0.25	0.14	0.106
	Sex	-0.11	0,77	0.886			
	Nickel (μg/L)	0.71	0.30	0.025			
Cholesterol	Age (years)	-1,88	1,14	0.113	0.27	0.17	0.077
	Sex	16,16	17,15	0.357			
	**Arsenobetaine (μg/L)**	**0,70**	**0,32**	**0.042**			

For sex variable, women are the reference group.

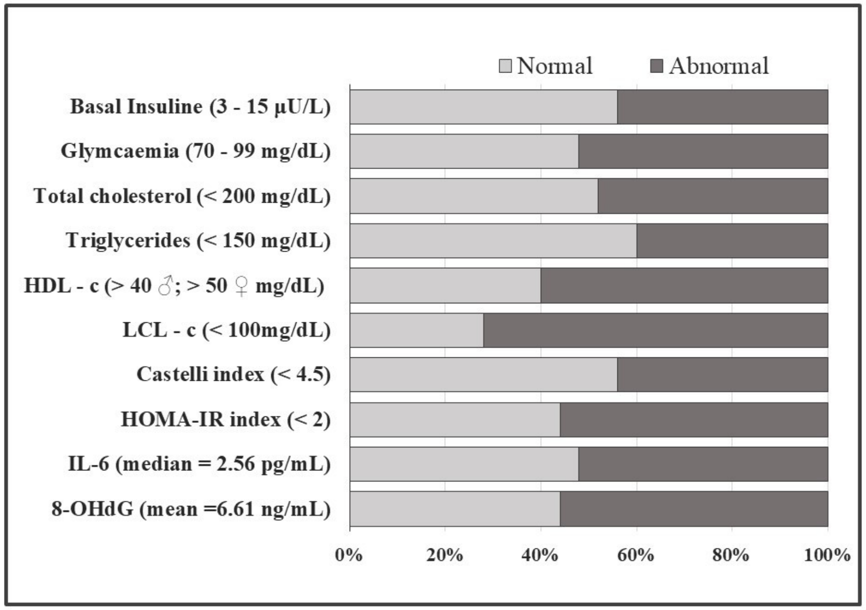

Supplementary Figure S1. Percentages of individuals with normal and altered biochemical parameters according to the National Health Survey (16, 21), or above and below of measurements of central tendency, according to their distribution, of the clinical parameters measured (median for IL-6: mean for 8-OHdG). See Table I for the dispersion values of the clinical parameters.
